# B-RAF^V600E^ Inhibitor Dabrafenib Attenuates RIPK3-Mediated Necroptosis and Promotes Functional Recovery after Spinal Cord Injury

**DOI:** 10.3390/cells8121582

**Published:** 2019-12-06

**Authors:** Takehiro Sugaya, Haruo Kanno, Michiharu Matsuda, Kyoichi Handa, Satoshi Tateda, Taishi Murakami, Hiroshi Ozawa, Eiji Itoi

**Affiliations:** 1 Department of Orthopaedic Surgery, Tohoku University School of Medicine, 1-1, Seiryo-machi, Aoba-ku, Sendai 980-8574, Japan; takehiro1224@gmail.com (T.S.); rin_miti_miti@yahoo.co.jp (M.M.); khanda@med.tohoku.ac.jp (K.H.); tateda3104@gmail.com (S.T.); taishi7891a@gmail.com (T.M.); itoi-eiji@med.tohoku.ac.jp (E.I.); 2 Department of Orthopaedic Surgery, Tohoku Medical and Pharmaceutical University, Faculty of Medicine, 1-15-1, Fukumuro Miyagino-ku, Sendai 983-8536, Japan; hozawa@tohoku-mpu.ac.jp

**Keywords:** spinal cord injury, RIPK3, dabrafenib, necroptosis, B-RAF

## Abstract

The receptor-interacting protein kinase 3 (RIPK3) is a key regulator of necroptosis and is involved in various pathologies of human diseases. We previously reported that *RIPK3* expression is upregulated in various neural cells at the lesions and necroptosis contributed to secondary neural tissue damage after spinal cord injury (SCI). Interestingly, recent studies have shown that the B-RAF^V600E^ inhibitor dabrafenib has a function to selectively inhibit RIPK3 and prevents necroptosis in various disease models. In the present study, using a mouse model of thoracic spinal cord contusion injury, we demonstrate that dabrafenib administration in the acute phase significantly inhibites RIPK3-mediated necroptosis in the injured spinal cord. The administration of dabrafenib attenuated secondary neural tissue damage, such as demyelination, neuronal loss, and axonal damage, following SCI. Importantly, the neuroprotective effect of dabrafenib dramatically improved the recovery of locomotor and sensory functions after SCI. Furthermore, the electrophysiological assessment of the injured spinal cord objectively confirmed that the functional recovery was enhanced by dabrafenib. These findings suggest that the B-RAF^V600E^ inhibitor dabrafenib attenuates RIPK3-mediated necroptosis to provide a neuroprotective effect and promotes functional recovery after SCI. The administration of dabrafenib may be a novel therapeutic strategy for treating patients with SCI in the future.

## 1. Introduction

The neurological outcome of spinal cord injury (SCI) is caused by the initial mechanical tissue damage and the consequent cellular and molecular events, known as secondary injury, that increase the lesion size in the injured spinal cord [1,2]. Secondary injury has been considered to be mainly caused by apoptosis occurring in the hours to weeks after SCI [3,4]. Over the past few decades, numerous studies have been conducted to identify effective therapeutic strategies to reduce secondary tissue damage following SCI. Most of these studies have mainly focused on the prevention of apoptosis [1]. However, no clinically useful therapeutic strategy that can inhibit secondary damage has yet been established.

Necroptosis, which was recently identified as another type of programmed cell death, is regulated by the caspase-independent pathway and has the morphological characteristics of necrosis [5,6]. The receptor-interacting protein kinase 3 (RIPK3) and phosphorylate mixed lineage kinase domain-like (MLKL) are critical mediators of necroptosis execution [7,8]. Necroptosis can be induced by different stimuli, including TNF-α, TRAIL, and FasL [9]. Various stimuli to cells induce the formation of necrotic complexes that contain RIPK1, TRADD, FADD, and caspase-8. Under certain conditions, such as caspase-8-inhibited conditions, RIPK1 and RIPK3 aggregate in microfilament-like complexes known as necrosomes. The phosphorylation of RIPK3 in the necrosome results in the recruitment and consequent phosphorylation of MLKL [10]. Recently, an increasing number of studies have provided evidence suggesting that necroptosis is involved in various human diseases [11]. Importantly, necroptosis plays important roles in pathological conditions in animal models of CNS diseases, such as Alzheimer’s disease, Parkinson’s disease, and amyotrophic lateral sclerosis [12]. In addition, necroptosis contributes to neural tissue damage in models of adult brain ischemia [13,14,15], neonatal hypoxia–ischemia [16], and traumatic brain injury [17]. We previously showed that the *RIPK3* expression was significantly upregulated in various neural cells at the lesion following SCI and that necroptosis contributed to neural tissue damage due to secondary injury [18]. It has also been reported that necroptosis is associated with lysosomal damage as well as endoplasmic reticulum stress in the injured spinal cord [19,20]. In addition, several studies have suggested that the RIPK1 inhibitor necrostatin-1 (nec-1) induces a neuroprotective effect and improves the locomotor recovery after SCI [21,22]. The RIPK1 inhibitor Nec-1 and several compounds inhibiting different pathways have been widely used in many experimental studies [9,23].

Dabrafenib is a B-RAF^V600E^ inhibitor that has been used as an anti-cancer drug for various human diseases [24], such as metastatic melanoma [25,26], non-small-cell lung cancer [27], colorectal cancer [28], and thyroid cancer [29]. Interestingly, a recent study revealed that the B-RAF^V600E^ inhibitor dabrafenib selectively inhibits RIPK3 [30]. Previous studies have shown that the administration of dabrafenib inhibits RIPK3 and decreases necrotic cell death in various disease models, both in vitro and in vivo [30,31,32,33]. Notably, in an acetaminophen-induced liver injury model, dabrafenib was found to inhibit RIPK3 on inducing hepatocyte cell death and to reduce the liver damage [30]. In addition, in an in vitro model of toxic epidermal necrolysis, dabrafenib prevented RIPK3-mediated MLKL phosphorylation and decreased cell death [33]. Furthermore, dabrafenib exerted a neuroprotective effect and reduced the infarct volume in a model of ischemic brain injury [31]. However, no study has investigated the therapeutic effect of dabrafenib on SCI.

In this study, we examine whether the administration of dabrafenib attenuates RIPK3-mediated necroptosis and secondary injury and consequently improves functional recovery following SCI using a mouse model of thoracic spinal cord contusion injury. Surprisingly, we found that dabrafenib administration in the acute phase significantly inhibited RIPK3-mediated necroptosis and reduced secondary neural tissue damage, such as demyelination, neuronal loss, and axonal damage, following SCI. Furthermore, the neuroprotective effect of dabrafenib dramatically enhanced the recovery of locomotor and sensory functions after SCI. Our findings are thus considered to provide evidence supporting a novel therapeutic strategy involving the inhibition of RIPK3-mediated necroptosis by dabrafenib.

## 2. Materials and Methods

### 2.1. Animals

In the present study, we used adult female C57BL/6J mice (10–12 weeks of age; Japan SLC, Inc., Shizuoka, Japan). The animals were maintained at the specific pathogen-free animal facilities of our institute, under a 12-h dark/12-h light cycle. The mice were housed at 4 or 5 per cage in a room kept at 24 °C with free access to water and food before and after surgery. All experimental procedures were approved by the Institutional Animal Care and Use Committee of Tohoku University (#2019-158). All efforts were made to minimize the number of animals used and to decrease their suffering.

### 2.2. Spinal Cord Injury

The animals were anesthetized with 4% sevoflurane. Laminectomy was performed at T10 to expose the dorsal surface of the spinal cord with the dura intact. The spinal cord contusion injury was made using a modified MASCIS Impactor [34,35,36]. A 10-g rod (tip diameter: 1.5 mm) was dropped from 3 mm onto the T10 segment. The rectal temperature was maintained at 37.0 ± 0.5 °C by a heating pad during surgery. After surgery, the bladders were expressed twice a day until spontaneous voiding began. In sham control mice, laminectomy was performed without SCI.

### 2.3. Dabrafenib Administration

Mice were randomly assigned to vehicle-treated and dabrafenib-treated groups. Dabrafenib (MedChemExpress, Monmouth Junction, NJ, USA) was suspended in an aqueous mixture of 0.5% hydroxypropyl methyl cellulose (HPMC) and 0.2% Tween 80 (Sigma-Aldrich, St. Louis, MO, USA). The suspended drug (200 µL) was administered by oral gavage to mice in the dabrafenib-treated group at 100 mg/kg of dabrafenib at 4, 24, and 48 h after SCI [30,37]. In the vehicle-treated mice, an equivalent volume of the vehicle was administrated by oral gavage, as described previously [30,37].

### 2.4. Locomotion

This study performed locomotor rating tests using the Basso mouse scale (BMS) score for 42 days following SCI [38]. The BMS score can be used to evaluate the recovery of locomotor function, including joint movements, stepping ability, coordination, and trunk stability. The BMS subscore was also assessed because some animals show improvements in specific aspects of locomotion that do not follow a typical pattern of recovery and, therefore, are not reflected in any change in the overall score on the BMS [38]. The animals were placed in a molded plastic open field, and well-trained investigators scored them on the BMS in a blinded manner. The BMS scores were averaged to determine a single value per mouse per test. The animals were placed individually in the open field for 4 min to confirm that all subjects consistently obtained the maximum score before SCI.

To further assess the improvement in the motor function, we performed the inclined plane (IP) test [39]. This test can determine the degree of muscle paralysis recovery by measuring the muscle strength of the lower limbs. In the IP test, the mice were placed horizontally on a board of the dedicated testing machine (sliding machine SN-453; Shinano, Tokyo, Japan), and the maximum angle of the board at which the animal could maintain its balance for 5 s was recorded. We analyzed the average of four trials in each mouse.

### 2.5. Mechanical Hypersensitivity

Using the von Frey method, the mechanical hypersensitivity of the planter hindpaws was determined weekly until 42 days following SCI. The von Frey test evaluates the cutaneous sensitivity to innocuous mechanical stimulation of both hindpaws using the up–down method [40] with 8 specifically calibrated the von Frey filaments (4.0, 2.0, 1.0, 0.6, 0.4, 0.16, 0.07, and 0.04 g; Muromachi Kikai Co., Ltd., Tokyo, Japan). The series of responses to the filaments was converted into a 50% withdrawal threshold, measured in grams [41]. To obtain a single value for each animals, the withdrawal threshold was calculated as the average of the left and right hindpaws.

### 2.6. Thermal Hypersensitivity

According to the Hargreaves method [42], the thermal hypersensitivity of the planter hindpaws was investigated. The animals were placed unrestrained in individual clear plastic compartments on an elevated glass floor. The Plantar Test Apparatus (Ugo Basile, Gemonio, Italy) was applied through the glass floor to the middle of the plantar surface of the hindpaws. A photocell automatically stopped the heat source and the timer when the mouse lifted its paw for assessing the latency to withdrawal from the heat source. A cut-off of 20 s was used in order to prevent tissue damage. To determine the final withdrawal latency, the average of three trials/paw was calculated.

### 2.7. Western Blot

To assess whether the dabrafenib administration can inhibit the RIPK3-mediated necroptosis, levels of the *RIPK3* expression in the spinal cord were analyzed using Western blot. The animals were sacrificed at three days following SCI, and then the spinal cords of 5 mm length containing the injured site were collected. The spinal cords were homogenized in lysis buffer, and the debris was removed by centrifugation. The proteins in the lysates were separated by SDS-polyacrylamide gel electrophoresis (PAGE) in 12% gels and then electrophoretically transferred to polyvinylidene difluoride membranes. The membranes were blocked for 1 h in TBST buffer (0.01 M Tris HCL, pH 7.5, 0.15 M NaCl, and 0.05% Tween 20) containing 3% milk and incubated with rabbit anti-RIPK3 antibodies (1:1000; PRS2283, Sigma-Aldrich) diluted in TBST buffer overnight at 4 °C. The membranes were then incubated with secondary antibodies linked to horseradish peroxidase (1:2000, P0448, DAKO, Glostrup, Denmark) for 1 h at room temperature. The immunoreactive bands were developed using enhanced chemiluminescence reagent (ECL; Amersham plc, Amersham, UK). Using a scanned densitometric analysis with the Image Lab software program, version 6.0 (Bio Rad Laboratories, Hercules, CA, USA), the band density was quantified. The quantity of the band density was normalized according to the level of β-tubulin (Developmental Studies Hybridoma Bank, Iowa City, IA, USA).

### 2.8. Tissue Preparation

At 3 or 42 days after SCI, the mice were overdosed with an intraperitoneal injection of 100 mg/kg sodium pentobarbital. The mice were transcardially perfused with normal saline, followed by 4% paraformaldehyde in 0.1 M phosphate-buffered saline (PBS) at pH 7.4. For immunohistochemical staining, the spinal cord segments containing the injured site were collected, post-fixed in the same fixative overnight at 4 °C, cryoprotected in 30% sucrose in PBS for 48 h at 4 °C, embedded in optimal-cutting temperature (OCT) compound (Thermo Scientific Ltd.), and cut on a cryostat into 15-µm-thick transverse sections. A total of 5 sequential sections at 500-µm intervals were collected, spanning a 2000-µm length of the spinal cord centered at the epicenter. These spinal cord sections were used for immunohistochemical staining and luxol fast blue staining in this study.

### 2.9. Immunostaining

Immunostainings for RIP3 and pMLKL were performed using the spinal cord sections obtained 3 days, and those for NeuN and RT97 were performed using sections obtained 42 days after SCI. The sections were washed in PBS for 15 min, after which they were washed with PBS containing 0.3% Tween for 10 min and blocked with 3% milk and 5% FBS in 0.01 M PBS for 2 h. The sections were incubated with rabbit anti-RIPK3 antibody (1:100; PRS2283, Sigma-Aldrich), rabbit anti-phospho-MLKL antibody (1:150; ab196436, Abcam, Cambridge, UK), mouse anti-NeuN antibody (1:100; MAB377, Millipore, Burlington, MA, USA) or mouse anti-RT97 antibody (1:30; AB528399, DSHB, Iowa City, IA, USA) diluted in PBS overnight at 4 °C. After rinsing with PBS, the sections were incubated with goat anti-rabbit IgG Alexa Fluor 488 secondary antibody (1:500; A11034, Invitrogen, Carlsbad, CA, USA), donkey anti-rabbit IgG Alexa Fluor 594 secondary antibody (1:500; A21207, Invitrogen), goat anti-mouse IgG Alexa Fluor 488 secondary antibody (1:500; A11001, Invitrogen), or donkey anti-mouse IgG Alexa Fluor 594 secondary antibody (1:500; A21203, Invitrogen) for 1 h at room temperature. The sections were mounted with Vectashield containing DAPI to label the nuclei (Vector Laboratories). The sections were stained at the same time in each experiment.

### 2.10. Counting of RIPK3-Positive Cells, pMLKL-Positive Cells, and NeuN-Positive Cells

The RIPK3-, pMLKL-, and NeuN-stained sections were scanned using a laser microscope (BX 51; Olympus, Tokyo, Japan). The scanned image of the transverse section was displayed on a monitor with a grid using the Photoshop CC 2018 software program, version 19 (Adobe, San Jose, CA, USA). Using a manual counter, RIPK3-, pMLKL-, and NeuN-positive cells in the section were counted. These positive cells were defined as cells double-labeled with DAPI and each anti-body. The sections at the epicenter and 500 µm rostral and caudal sections were chosen for each animal. The number of positive cells in each section and the sum of the numbers in all three sections for each animal were compared between the dabrafenib-treated and vehicle-treated mice.

### 2.11. Quantitative Analyses of the Neurofilament Staining

The quantitative assessment of the RT97 immunoreactivity was performed by measuring the immunodensity. The immunodensity was measured using the Image J software program (version 1.50i, National Institutes of Health, Maryland, MD, USA). The threshold optical density that best discriminated the staining from the background was obtained for each stain. The threshold setting was applied consistently and automatically in each image. According to the previous studies [18,36], total pixels of the stained area were measured and expressed as the immunodensity. The sections at the epicenter and 500 µm rostral and caudal sections were chosen for each animal. The immunodensity in each section were then compared between the dabrafenib-treated and vehicle-treated animals.

### 2.12. Luxol Fast Blue Staining

To assess the white matter sparing, the serial transverse sections around the epicenter 42 days after SCI were stained with luxol fast blue. A total of 5 sequential sections spanning 2000 µm of spinal cord length, which included two sections, both rostral and caudal to the section at the epicenter, were assessed. The section with the least amount of luxol fast blue stain was selected as the lesion epicenter. The images of the stained sections were captured, and the spared white matter area of the spinal cord was analyzed using the Image J 1.50i software program (National Institutes of Health). After performing luxol fast blue staining, the spared white matter appeared blue and isocellular, as seen in healthy neuronal tissue. The damaged or degenerated white matter appeared to be either blanched or replaced by scar tissue, showing clusters of cells with prominent basophilic nuclei [35,43]. The percentage of the spared white matter area was calculated from the proportion of the spared white matter area to the total cross-sectional area of the whole cord. The percentage of the spared white matter area was compared between the dabrafenib-treated and vehicle-treated mice in each section.

### 2.13. Propidium Iodide Labeling

Propidium iodide (PI) labeling was performed in order to detect the plasmalemma permeability, which is a hallmark of necrotic cell death, in the injured spinal cord at three days after SCI as previously described [17,18,44,45]. In brief, the PI solution (1 mg/mL; Sigma-Aldrich) was injected intraperitoneally at a dose of 1 mg/kg body weight at 1 h before sacrifice. The mice were then transcardially perfused, and the spinal cords were collected and sectioned as described above and mounted with Vectashield containing DAPI. Using a fluorescence microscope (BX 51; Olympus), the PI labeling was detected using emission and excitation wavelengths of 550 nm. The PI-positive cells were counted using the Image J 1.50i software program (National Institutes of Health). The threshold setting was applied consistently and automatically in each image. The number of PI-positive cells in each section and the sum of the numbers in the three sections for each animal were compared between the dabrafenib-treated and vehicle-treated mice.

### 2.14. Electrophysiology

For the electrophysiological assessment of the spinal cord function, the measurement of motor evoked potentials (MEPs) is commonly used to evaluate the preservation of the central motor pathway after SCI [46]. The MEPs correlate with the severity of neural tissue damage, including demyelination and axonal damage, as well as the locomotor function after SCI [47,48]. In this study, we measured the MEPs to evaluate the neuronal circuit from the upper cervical cord to the hindlimbs at 42 days after SCI. The MEPs elicited by transcranial electrical stimulation were measured using an electromyogram of the gastrocnemius muscle in the hindlimb [49]. The MEPs were evoked and recorded by electromyography and an evoked potential response unit (Neuropack MEB2300; Nihon Koden, Tokyo, Japan). Mice were given an intraperitoneal injection of ketamine (80 mg/kg) and xylazine (16 mg/kg). A pair of needle stimulating electrodes were placed subcutaneously at 3 mm on each side of the vertex of the skull, and needle electrodes were placed in one of the hindlimbs. The active electrode was placed in the muscle belly, and the reference electrode was placed near the distal tendon of the muscle in the limb. The ground electrode was placed subcutaneously between the coil and the recording electrodes. Short trains of 3 square-wave stimuli of 0.5 ms duration with an interstimulus interval of 2 ms were delivered. The MEP onset latency was measured as the length of time between the stimulus and the onset of the first increase in the amplitude of the electromyography response [47,48,50].

### 2.15. Statistics

The significance of differences in the number of RIP3, pMLKL and NeuN-positive cells, the immunodensity of RT97 staining, the band densities obtained from the Western blot analyses, the number of PI-positive cells, the white matter area and the latencies of MEPs were analyzed using the Mann–Whitney U test. The significance of differences in the BMS score, subscore, mechanical hypersensitivity, and thermal hypersensitivity was determined by the Mann–Whitney U test at each point. In all analyses, a value of *p* < 0.05 was considered to be statistically significant. All data are presented as the means ± standard deviation. All statistical analyses were performed using the Statcel 4 software program (OMS Publishing Inc., Tokyo, Japan).

## 3. Results

### 3.1. Dabrafenib Improves Locomotor Function after SCI

**Figure 1:**
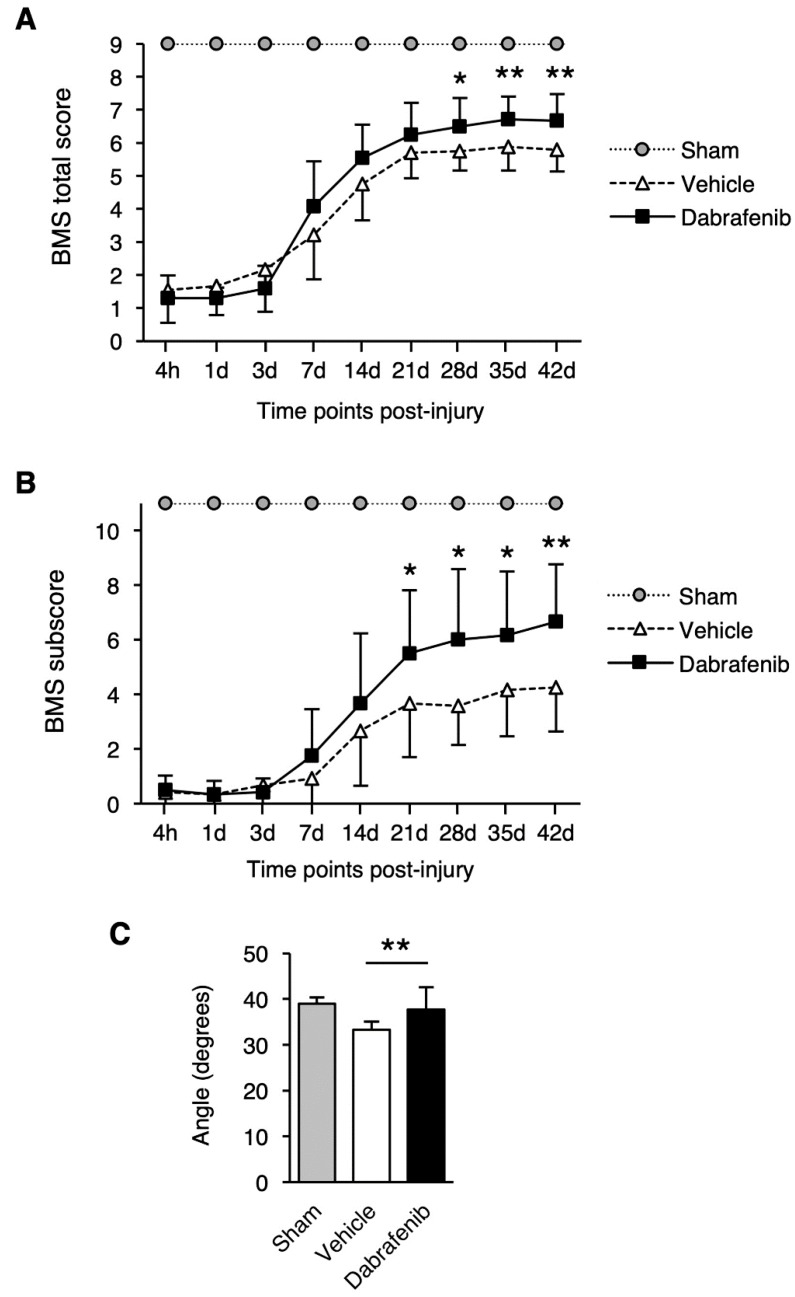
Recovery of locomotor function following SCI in vehicle-treated mice and dabrafenib-treated mice. The BMS score (**A**) and BMS subscore (**B**) were measured from 4 h to 42 days after SCI. (**A**) The dabrafenib-treated mice had significantly higher total BMS scores than the vehicle-treated mice from days 28 to 42. (**B**) The BMS subscores were also significantly higher in the dabrafenib-treated mice than in the vehicle-treated mice from days 21 to 42. (**C**) The IP angle in the dabrafenib-treated mice was significantly higher than that in the vehicle-treated mice. All data are presented as mean ± SD. n = 10 for vehicle and dabrafenib groups, n = 5 for sham group; * *p* < 0.05, ** *p* < 0.01.

To evaluate the effect of dabrafenib treatment on the recovery of the locomotor function, the total scores and subscores of the BMS were measured for 42 days following SCI (Figure 1A,B). Remarkably, we found that the total BMS scores were consistently higher in the dabrafenib-treated animals compared with the vehicle-treated animals from 7 to 42 days after injury. Furthermore, the dabrafenib-treated animals had significantly higher total BMS scores than the vehicle-treated animals from 28 to 42 days (Figure 1A). Similarly, in the BMS subscore, the dabrafenib-treated group showed significantly better locomotor function than the vehicle-treated group from 21 to 42 days after injury (Figure 1B). Notably, 8 out of 12 animals in the dabrafenib-treated group were able to keep their hindpaws parallel while stepping at 42 days after injury. In contrast, 9 out of 12 vehicle-treated mice could not keep their hindpaws parallel. In addition, three animals in the dabrafenib-treated group showed normal trunk stability or only mild trunk instability. In contrast, all of the vehicle-treated mice showed severe trunk instability, such as leaning, waddling, or near-collapse of the hindlimbs, at 42 days after injury.

To further assess the improvement in the locomotor function, the IP test was performed. The IP test determines the degree of muscle paralysis recovery by measuring the maximum angle of the board at which the animal is able to maintain its balance. In the results of the IP test, the IP angle in the dabrafenib-treated mice was significantly higher than that in the vehicle-treated mice (Figure 1C), suggesting that the recovery of muscle paralysis was better in the dabrafenib-treated mice. Taken together, the behavioral analysis of the locomotor function suggested that the administration of dabrafenib in the acute phase after injury provides a therapeutic effect that reduces locomotor impairment following SCI.

### 3.2. Dabrafenib Attenuates Mechanical and Thermal Hypersensitivity in the Hindpaws Following SCI

**Figure 2:**
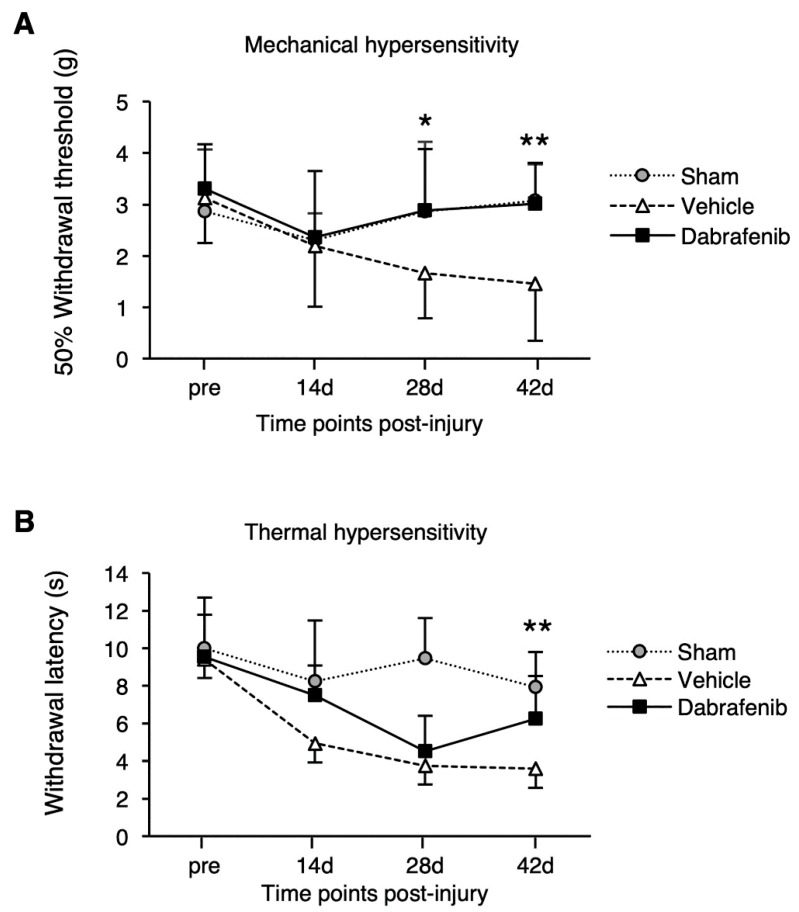
Mechanical and thermal hypersensitivity in the hindpaws for 42 days after SCI. (**A**) The withdrawal threshold using the von Frey test for assessing the mechanical hypersensitivity. The dabrafenib-treated mice showed significantly better improvement of the mechanical hypersensitivity than the vehicle-treated mice at 28 and 42 days. (**B**) The withdrawal latency using Hargreaves’ test for assessing the thermal hypersensitivity. The dabrafenib-treated mice showed significantly better improvement of thermal hypersensitivity compared to the vehicle-treated mice at 42 days. All data are presented as mean ± SD. n = 10 for vehicle and dabrafenib groups, n = 5 for sham group; * *p* < 0.05, ** *p* < 0.01.

To address the effect of dabrafenib on the recovery of the sensory function after SCI, the mechanical and thermal allodynia in the hindlimbs were evaluated (Figure 2). The von Frey test measures the withdrawal threshold while applying mechanical stimulation to the hindpaws of mice. The results of the von Frey test revealed that the withdrawal threshold in the dabrafenib-treated animals was consistently higher than that in the vehicle-treated animals from 28 to 42 days after injury. Furthermore, the dabrafenib-treated animals showed significantly better improvement of the mechanical hypersensitivity than the vehicle-treated animals at 28 and 42 days (Figure 2A). Next, we performed the Hargreaves test to measure the withdrawal latency of the hindlimbs during a brief noxious heat stimulus to the plantar hindpaws of mice. The results showed that the withdrawal latency in the dabrafenib-treated animals was longer than that in the vehicle-treated animals from 14 to 42 days after injury. The dabrafenib-treated animals showed significantly better improvement of thermal hypersensitivity at 42 days (Figure 2B). These findings indicate that dabrafenib treatment may ameliorate the mechanical and thermal hypersensitivities following SCI.

### 3.3. Dabrafenib Inhibits the Activation of RIPK3-MLKL Signaling Pathway after SCI

**Figure 3:**
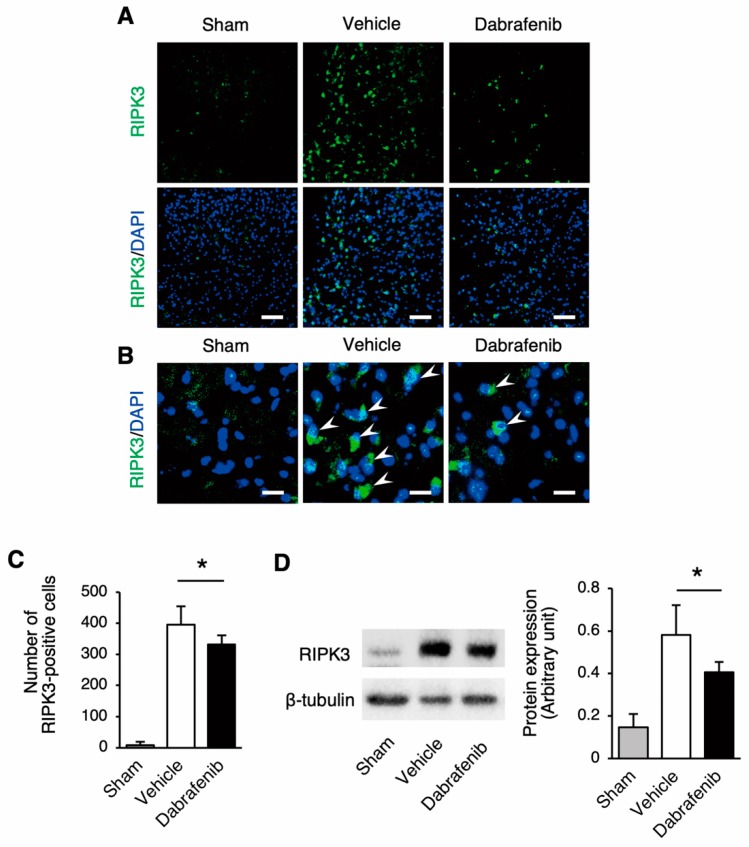
*RIPK3* expression in damaged neural tissue after SCI. (**A**) The immunohistochemical staining of RIPK3 at 3 days after injury. Representative sections in the dorsal horn at the epicenter showed that the population of RIPK3 expressing cells markedly decreased in the dabrafenib-treated mice than in the vehicle-treated mice. Scale bars: 100 µm. (**B**) On higher magnification, RIPK3-positive cells were observed in the dabrafenib-treated mice and the vehicle-treated mice (arrowheads). Scale bars: 25 µm. (**C**) The number of RIPK3-positive cells was significantly lower in the dabrafenib-treated mice than in the vehicle-treated mice. (**D**) A Western blot analysis revealed that the expression of RIPK3 protein in the spinal cord at 3 days after injury was significantly decreased in the dabrafenib-treated mice than in the vehicle-treated mice. All data are presented as mean ± SD. n = 6 for vehicle and dabrafenib groups, n = 5 for sham group; * *p* < 0.05.

To address whether dabrafenib inhibits the RIPK3-MLKL signaling pathway after SCI, the RIPK3 protein expression in the spinal cord was examined using immunohistochemistry and Western blot (Figure 3). The immunohistochemical analysis of RIPK3 showed that the cells expressing RIPK3 were increased in the injured spinal cords of the dabrafenib-treated mice and the vehicle-treated mice compared to the uninjured cord of the sham control mice (Figure 3A,B). Importantly, the population of RIPK3 expressing cells markedly decreased in the dabrafenib-treated mice than in the vehicle-treated mice. The number of RIPK3-positive cells was significantly lower in the dabrafenib-treated mice than in the vehicle-treated mice (Figure 3C). Furthermore, the Western blot analysis revealed that the expression of RIPK3 protein in the injured spinal cord was significantly decreased in the dabrafenib-treated mice compared to the vehicle-treated mice (Figure 3D).

**Figure 4:**
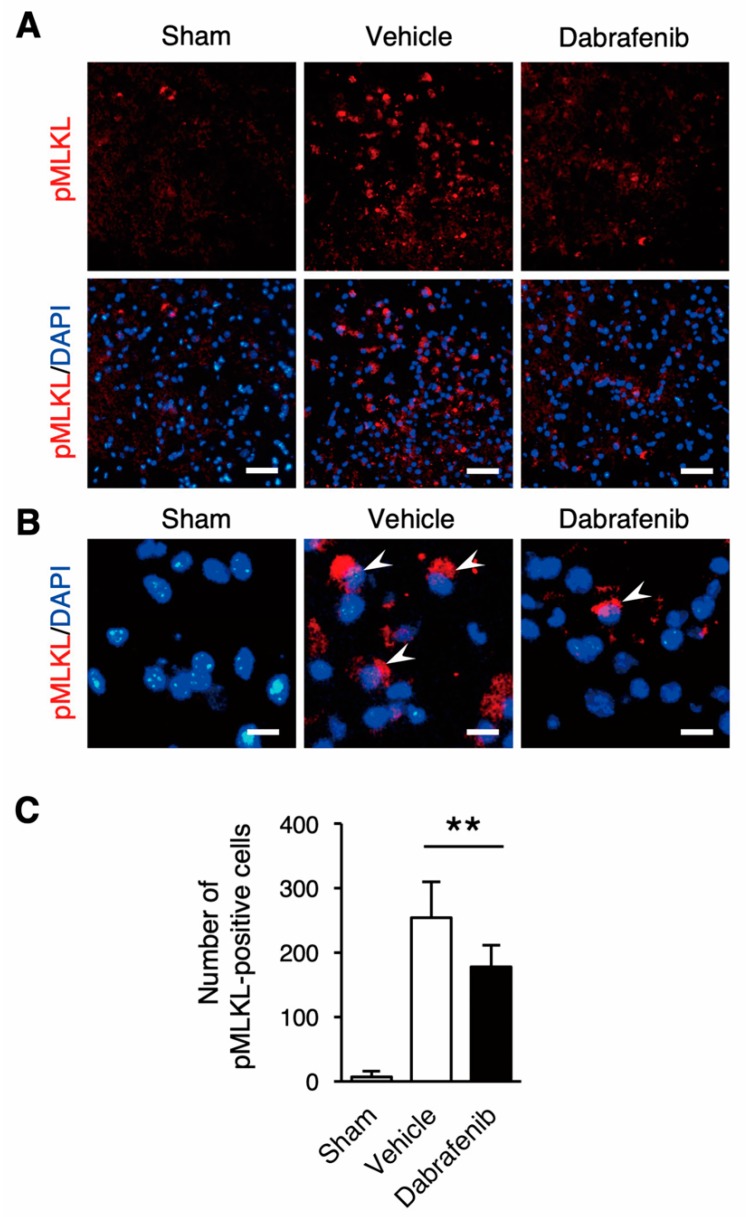
Immunostaining of pMLKL at 3 days after SCI. (**A**) In representative sections in the dorsal horn at the epicenter, the pMLKL-positive cells decreased in the dabrafenib-treated mice compared to the vehicle-treated mice. Scale bars: 100 µm. (**B**) On higher magnification, pMLKL-positive cells were observed in the dabrafenib-treated mice and the vehicle-treated mice (arrowheads). Scale bars: 25 µm. (**C**) The number of pMLKL-positive cells was significantly lower in the dabrafenib-treated mice than in the vehicle-treated mice. All data are presented as mean ± SD. n = 6 for vehicle and dabrafenib groups, n = 5 for sham group; ** *p* < 0.01.

Next, to investigate an alternation in the activation of MLKL following dabrafenib administration, we performed immunohistochemical analysis of phosphorylated-MLKL (pMLKL) using the spinal cord sections (Figure 4). In the immunohistochemical analysis, the cells expressing pMLKL were increased in the injured spinal cords compared to the uninjured spinal cord (Figure 4A,B). Importantly, we found that the number of pMLKL-positive cells was significantly lower in the dabrafenib-treated mice compared to the vehicle-treated mice (Figure 4C). These findings indicate that dabrafenib helps to suppress the activation of the RIP3-MLKL pathway, which can induce necroptosis in the injured spinal cord.

### 3.4. Administration of Dabrafenib Reduces Necrotic Cell Death in the Injured Spinal Cord

**Figure 5:**
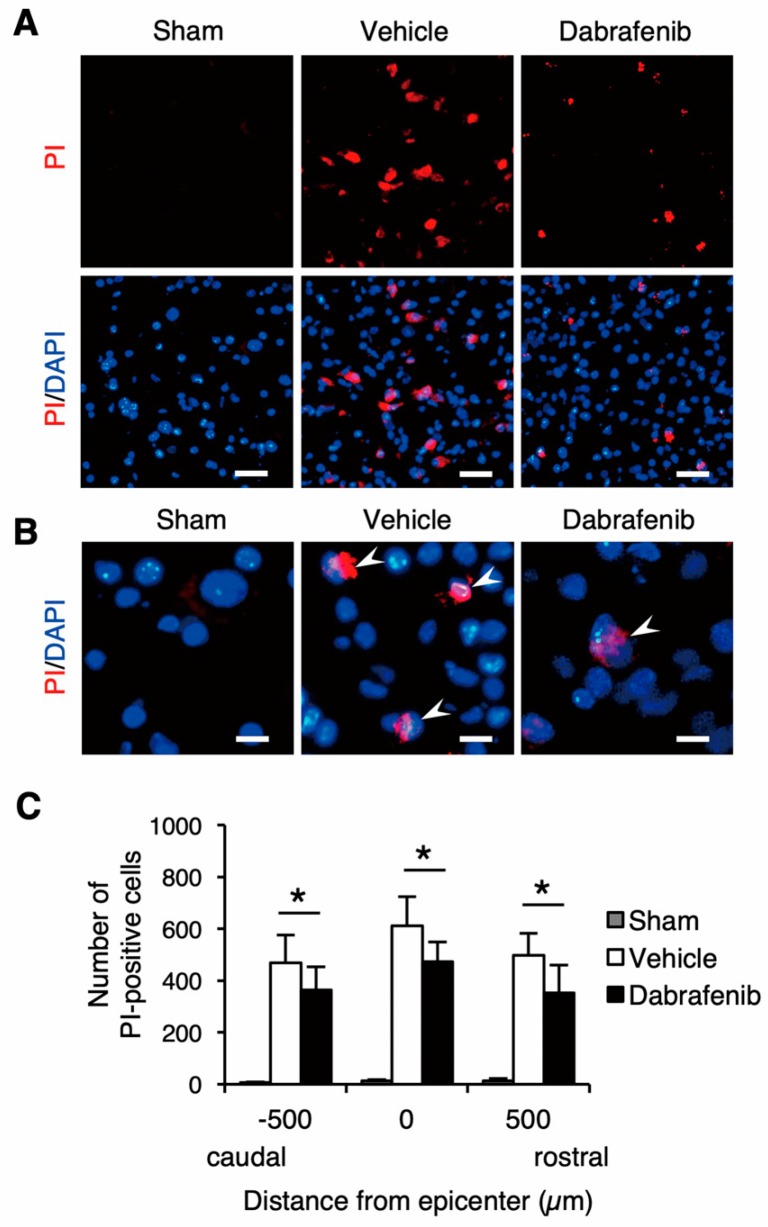
Assessment of necrotic cell death using propidium iodide (PI)-labeling at 3 days after SCI. (**A**) Representative sections in the ventral horn at the epicenter shows that the PI-positive cells decreased in the dabrafenib-treated mice compared to the vehicle-treated mice. Scale bars: 100 µm. (**B**) Magnified images showed PI-positive cells (arrowheads). Scale bars: 25 µm. (**C**) The numbers of PI-labeled cells were significantly lower in the dabrafenib-treated mice than in the vehicle-treated mice at 500 µm caudal and rostral from the epicenter and at the epicenter. All data are presented as mean ± SD. n = 6 for vehicle and dabrafenib groups, n = 3 for sham group; * *p* < 0.05.

In order to assess the necrotic cell death in the damaged neural tissue after SCI, we evaluated the PI labeling in the injured spinal cord (Figure 5). Interestingly, we found that the number of PI-labeled cells was decreased in the dabrafenib-treated mice compared to the vehicle-treated mice in the injured spinal cord (Figure 5A,B). The number of PI-labeled cells was significantly lower in the dabrafenib-treated mice than in the vehicle-treated mice at 500 µm rostral and caudal from the epicenter and at the epicenter (Figure 5C). These observations suggest that the administration of dabrafenib may reduce necrotic cell death via the inhibition of RIPK3-MLKL activation in the injured spinal cord.

### 3.5. Dabrafenib Improves White Matter Sparing after SCI

**Figure 6:**
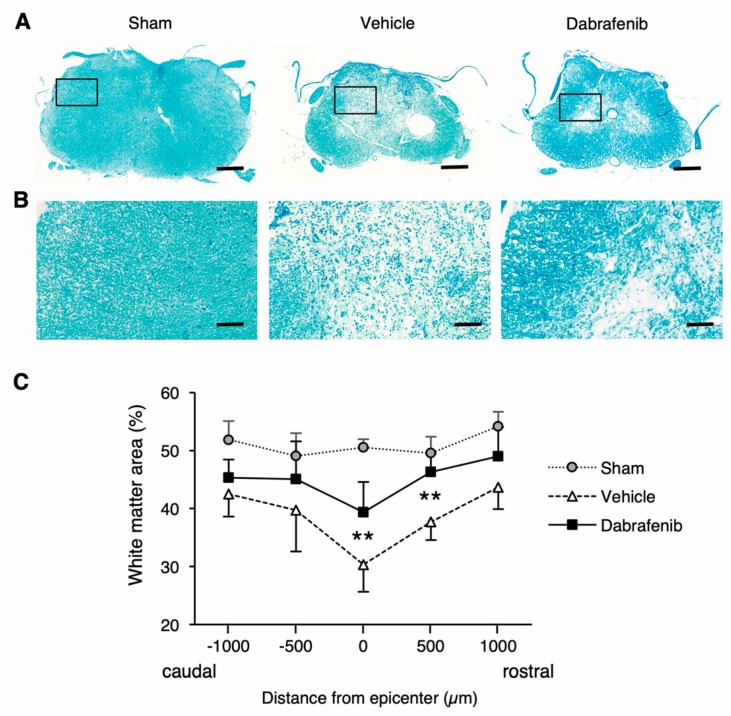
White matter staining with luxol fast blue at 42 days after SCI. (**A**) The areas of spared white matter in the dabrafenib-treated mice were markedly larger than in the vehicle-treated mice in the representative sections around the epicenter. Scale bars: 500 µm. High-magnification views of black boxes in (**A**) are shown in (**B**). (**B**) In higher magnification images, diffuse microcystic cavitation and clusters of cells with basophilic nuclei were more evident at the lesion of the spinal cord in the vehicle-treated mice than in the dabrafenib-treated mice. Scale bars: 100 µm. (**C**) The percentage of spared white matter area in the dabrafenib-treated mice was significantly larger than in the vehicle-treated mice and at the epicenter and 500 µm rostral from the epicenter. All data are presented as mean ± SD. n = 7 for vehicle and dabrafenib groups, n = 5 for sham group; ** *p* < 0.01.

Given that dabrafenib actually inhibits the activation of RIPK3-MLKL and reduces necrotic cell death in the injured spinal cord, we reasoned that the administration of dabrafenib might also reduce secondary neural tissue damage, such as demyelination, neuronal loss, and axonal degeneration, following SCI. To assess the demyelination, the area of spared white matter in the injured spinal cord were compared between the dabrafenib-treated and the vehicle-treated animals using luxol fast blue staining (Figure 6). Notably, we found that the areas of spared white matter in the dabrafenib-treated mice were markedly larger than in the vehicle-treated mice in the sections around the epicenter (Figure 6A). Consistently, a quantitative analysis revealed that the percentage of spared white matter area in the dabrafenib-treated animals was significantly larger than in the vehicle-treated animals at 500 µm rostral from the epicenter and at the epicenter (Figure 6C). Higher magnification images (Figure 6B) showed that diffuse microcystic cavitation and clusters of cells with basophilic nuclei were more evident at the lesion of the spinal cord in the vehicle-treated mice than in the dabrafenib-treated mice, suggesting that vacuolar degeneration of the white matter and pronounced inflammatory infiltration were decreased by dabrafenib.

### 3.6. Neuronal Loss and Axonal Damage at Lesion Site are Reduced by Dabrafenib

**Figure 7:**
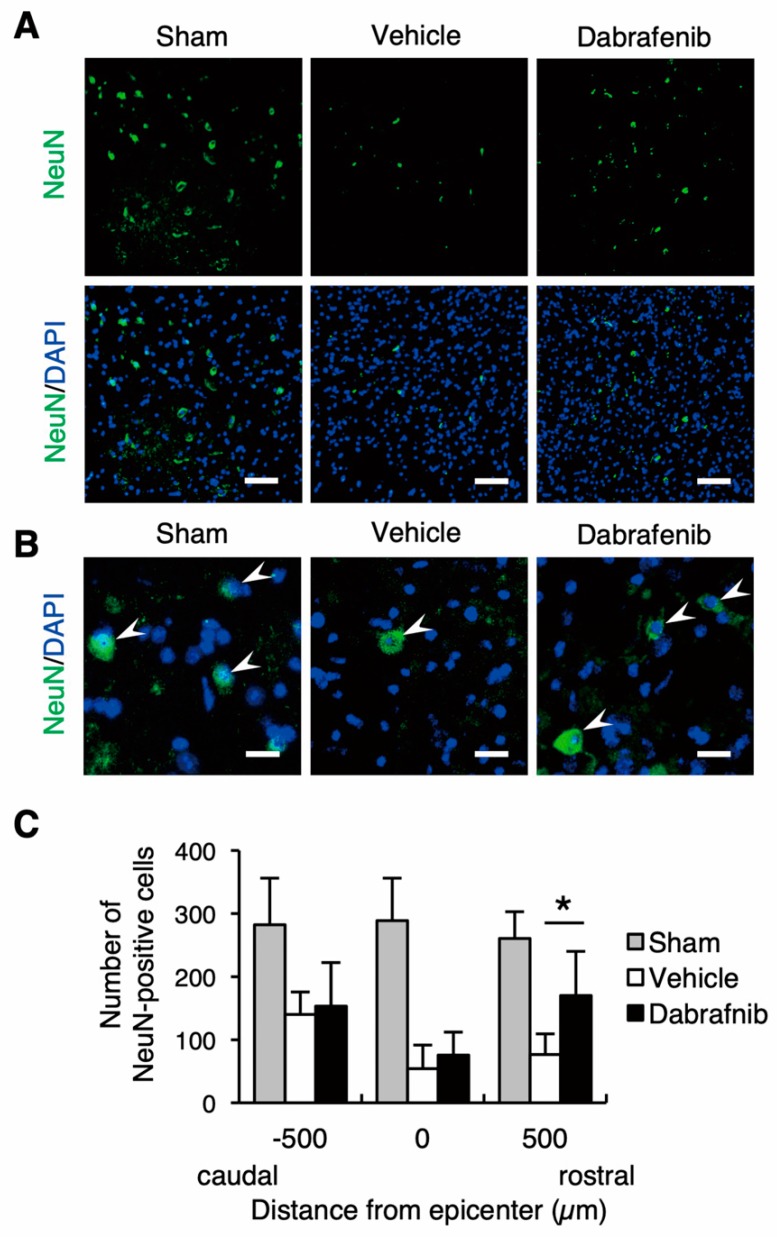
The immunohistochemical staining of NeuN at 42 days after SCI. (**A**) Representative sections in the ventral horn at the epicenter shows that NeuN-positive cells more evidently decreased in the vehicle-treated mice than in the dabrafenib-treated mice. Scale bars: 100 µm. (**B**) Magnified images showed NeuN-positive cells (arrowheads). Scale bars: 25 µm. (**C**) The number of NeuN-positive cells in the dabrafenib-treated mice was significantly larger than in the vehicle-treated mice at 500 µm rostral from the epicenter. All data are presented as mean ± SD. n = 7 for vehicle and dabrafenib groups, n = 5 for sham group; * *p* < 0.05.

To further address the neuronal loss after SCI, we performed immunostaining of NeuN using the spinal cord sections. The immunohistochemical analysis showed that the number of NeuN-positive cells was lower in the injured spinal cord than in the uninjured spinal cord of the sham control mice. Importantly, the decrease in NeuN-positive cells was more evident in the vehicle-treated mice than in the dabrafenib-treated mice (Figure 7A,B). In the NeuN-positive cells counting, the number of NeuN-positive cells in the dabrafenib-treated mice was significantly larger than in the vehicle-treated mice at 500 µm rostral from the epicenter (Figure 7C). These findings suggest that dabrafenib administration may attenuate the neuronal loss at the lesion site.

**Figure 8:**
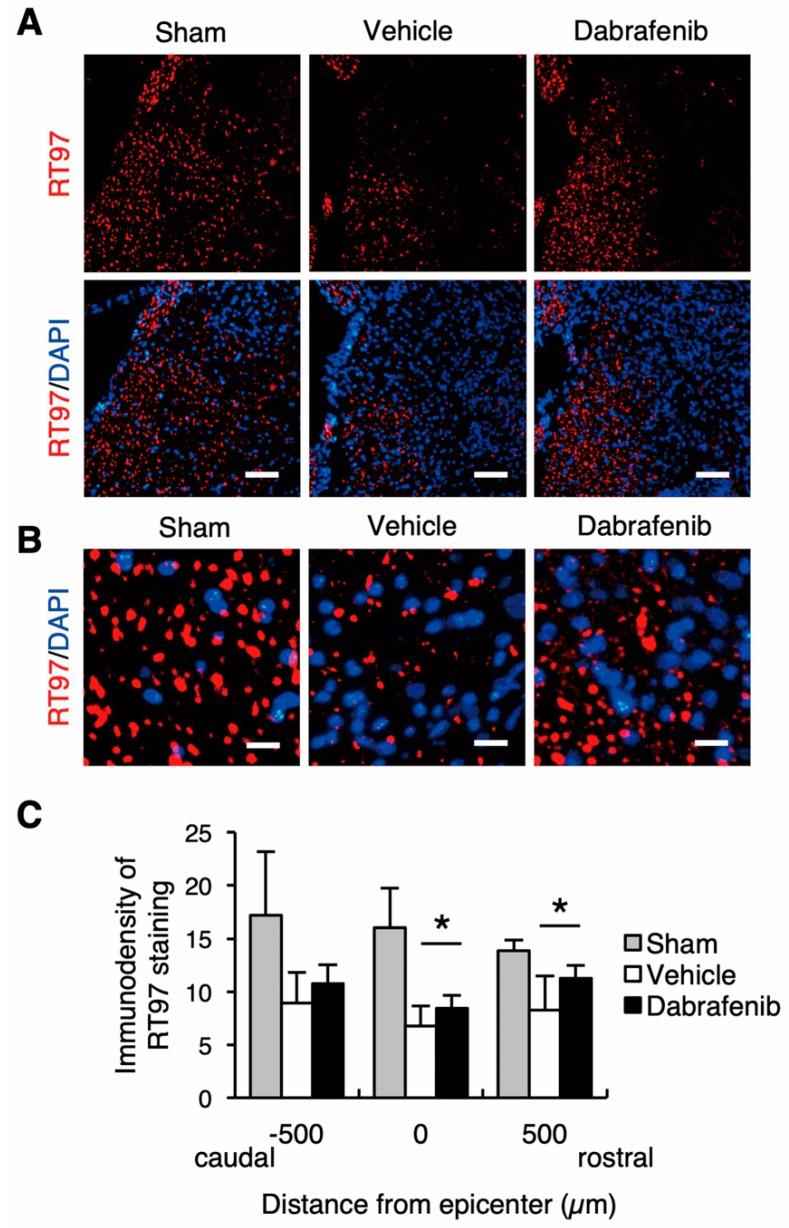
The immunohistochemical staining of RT97 at 42 days after SCI. (**A**) Representative sections in the dorsal horn at the epicenter. Scale bars: 100 µm. (**B**) Magnified images showed RT97-positive axons. Scale bars: 25 µm. (**C**) The immunodensity of RT97-positive axons was significantly higher in the dabrafenib-treated mice than in the vehicle-treated mice at 500 µm rostral from the epicenter and at the epicenter. All data are presented as mean ± SD. n = 7 for vehicle and dabrafenib groups, n = 5 for sham group; * *p* < 0.05.

To investigate the axonal damage, we also conducted immunostaining for neurofilament using the anti-RT97 antibody. The RT97-stained axonal fibers were decreased following injury compared with the uninured spinal cord (Figure 8). Importantly, the number of the RT97-stained axonal fibers observed at the lesion was markedly larger in the dabrafenib-treated mice than in the vehicle-treated mice (Figure 8A,B). Furthermore, the immunodensity of the RT97-stained area was significantly higher in the dabrafenib-treated mice than in the vehicle-treated mice at 500 µm rostral from the epicenter and the epicenter (Figure 8C). Therefore, these results suggested that the axonal damage in the injured spinal cord may be decreased by the administration of dabrafenib. Collectively, the results of the present study suggest that the dabrafenib administration in the acute phase may reduce secondary neural tissue damage, including demyelination, neuronal loss, and axonal damage, following SCI. The reduction in necrotic cell death via the inhibition of the RIPK3-MLKL signaling pathway by dabrafenib may provide a neuroprotective effect to the injured spinal cord.

### 3.7. Dabrafenib Helps Preserve the Central Motor Pathway and Physiological Function in the Injured Spinal Cord

**Figure 9:**
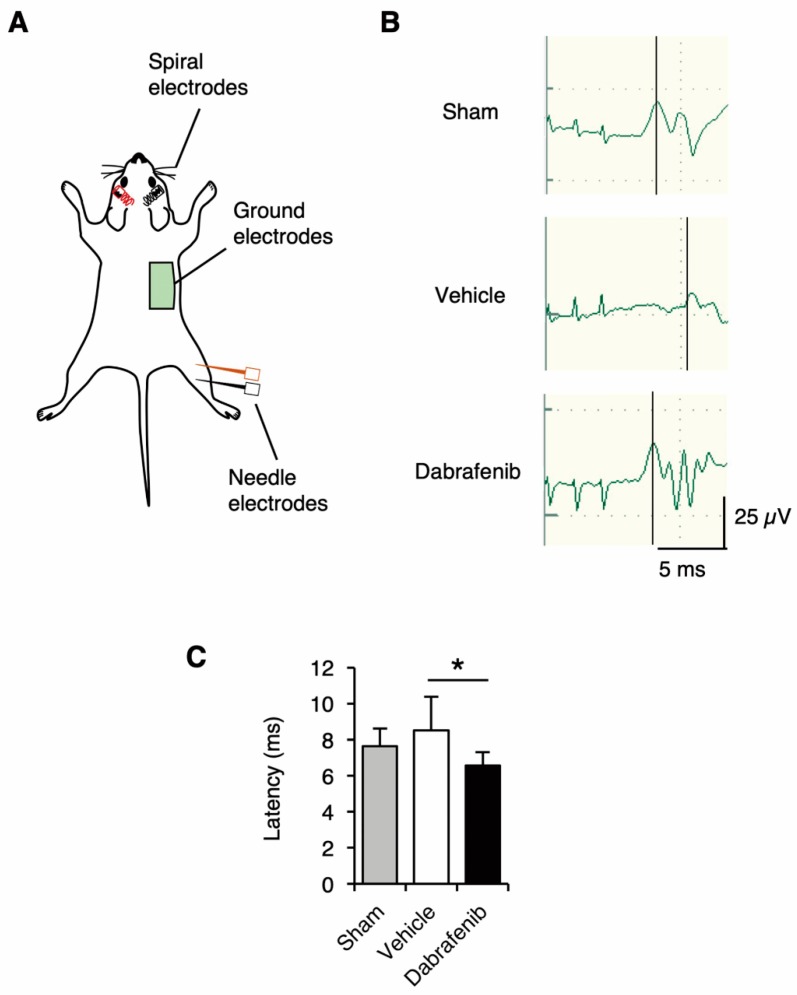
The electrophysiological function of the spinal cord at 42 days after SCI. (**A**) In the measurement of MEPs, spiral electrodes were placed at 3 mm on each side of the vertex of the skull, and needle electrodes were placed in right hindlimbs. The ground electrode was placed in the trunk. (**B**) The representative MEP data from each experimental group of mice. (**C**) The elongation of the latency of MEPs after injury was restored by dabrafenib treatment. All data are presented as mean ± SD. n = 8 for each group; * *p* < 0.05.

Given that the neural tissue damage and locomotor impairment were shown to be reduced by the administration of dabrafenib, we hypothesized that dabrafenib treatment might also ameliorate the electrophysiological dysfunction of the spinal cord following injury. We, therefore, assessed the electrophysiological function of the spinal cord using MEPs at 42 days after SCI (Figure 9A,B). The results showed that the latency of MEPs in the vehicle-treated mice was elongated compared to the sham control mice (Figure 9B,C). Notably, we observed that the elongation of the latency of MEPs after injury was restored by dabrafenib treatment. The latency of the MEPs in the dabrafenib-treated mice was significantly shorter than that in the vehicle-treated mice (Figure 9C). These findings suggest that the neuroprotective effect of dabrafenib may help preserve the central motor pathway and consequently attenuate the degree of electrophysiological dysfunction in the injured spinal cord.

## 4. Discussion

Substantial similarities are known to exist among the kinase domains of many human kinases. Recently, a study demonstrated that the kinase domains of RIPK1 and RIPK3 display a high degree of sequence similarity with the kinase domain of B-RAF [51]. This similarity suggests that inhibitors against the oncogenic kinase B-RAF may also inhibit the kinase activities of RIPK1 and/or RIPK3. Indeed, previous studies have reported that B-RAF inhibitors, such as dabrafenib, vemurafenib, and sorafenib, that are FDA-approved anti-cancer drugs, can block RIPK3 [30,52]. Interestingly, it was reported that the B-RAF^V600E^ inhibitor dabrafenib selectively inhibited RIPK3 and decreased the RIPK3-mediated phosphorylation of MLKL in various types of cells in vitro [30,33,53]. In addition, dabrafenib was found to disrupt the interaction between RIPK3 and MLKL and, consequently, to reduce necroptosis in an acetaminophen-induced liver injury model [30]. Similarly, dabrafenib also inhibited the RIPK3-mediated phosphorylation of MLKL and decreased necroptosis in the skin lesions of a toxic epidermal necrolysis model [33]. These data indicate that dabrafenib may potentially be an effective drug for the treatment of diseases related to RIPK3-mediated necroptosis. We previously reported that the *RIPK3* expression was upregulated at the lesion site and that necroptotic cell death contributed to secondary neural tissue damage after SCI [18]. In the current study, we further demonstrate that dabrafenib acts as an inhibitor of RIPK3-dependent cell death in an SCI model. Importantly, dabrafenib treatment significantly reduced the expressions of *RIPK3* and *pMLKL* in the injured spinal cord. Furthermore, the administration of dabrafenib significantly decreased the number of PI-positive cells at 3 days after SCI. Taken together, these findings suggest that the B-RAF^V600E^ inhibitor dabrafenib inhibited RIPK3-MLKL signaling and thereby prevented necrotic cell death in the damaged neural tissue after SCI.

Previous studies have suggested that necroptosis is involved in the pathology of CNS diseases and trauma [11,12]. The inhibition of the necroptosis may, therefore, improve the pathological conditions in the CNS. We previously reported that the upregulation of the RIPK3 expression following SCI was observed starting at 24 h, peaking at 3 days, and lasting for 21 days [18]. The time course of the *RIPK3* expression is very similar to that of secondary injury after SCI [3,4,54]. Our previous study also observed the increased expression of *RIPK3* in neurons, astrocytes, and oligodendrocytes in the damaged neural tissue after SCI, suggesting that necroptosis can occur in various neural cells in the injured spinal cord [18]. These observations imply that RIPK3-mediated necroptosis may be involved in different types of pathologies in secondary injury, such as neuronal damage, axonal degeneration, and demyelination. Interestingly, in the current study, we found that the administration of dabrafenib prevented RIPK3-mediated necroptosis and consequently attenuated neuronal loss following SCI. In addition, demyelination and axonal damage in the injured spinal cord were significantly reduced in the dabrafenib-treated animals. These findings suggest that dabrafenib treatment prevents necroptosis in different types of neural cells and consequently provides a neuroprotective effect that improves various pathological conditions following SCI.

Many studies have suggested that the inhibition of necroptosis produces a therapeutic effect that ameliorates the functional outcome in various disease models. Necrostatin-1 reduces necrotic cell death and the infarct size and preserves the cardiac function in a myocardial infarction model [55]. In a renal ischemia/reperfusion injury model, the inhibition of necroptosis reduces organ damage and improves the kidney function [56]. Necrostatin-1 protects photoreceptors from cell death and improves the functional outcome after experimental retinal detachment in rats [57]. In CNS injury models, necrostatin-1 reduces necrotic cell death and brain tissue damage and improves the spatial memory and motor function after traumatic brain injury [17]. Furthermore, the inhibition of RIPK1 by a specific chemical inhibitor or genetic knockdown also attenuates secondary brain injury after intracerebral hemorrhaging [58]. Recently, several studies have suggested that necroptosis can influence the locomotor function after SCI [22,59]. Importantly, the present study demonstrates for the first time that dabrafenib treatment reduces RIPK3-mediated necroptosis, which causes secondary injury, and improves the recovery of locomotor and sensory functions following SCI. The dabrafenib-treated animals showed a significantly better recovery of locomotion, as evaluated using the BMS score as well as IP testing, than vehicle-treated animals. The electrophysiological data of MEPs ascertained that the motor recovery of the dabrafenib-treated mice was markedly enhanced. Moreover, the dabrafenib significantly reduced sensory dysfunction, such as mechanical and thermal hypersensitivities. These results indicate that the administration of dabrafenib has therapeutic potential to enhance functional recovery after SCI. These improvements may have resulted from the therapeutic effect of dabrafenib to decrease secondary neural tissue damage, such as neuronal loss, axonal damage, and demyelination.

Drug repositioning of compounds that have already been approved by the FDA has advantages for the development of new drugs. There is a growing interest in drug repurposing because the development and approval of new drugs are associated with a costly and demanding process that also carries a high risk of failure. The advantage of drug repurposing resides in the fact that the development track may avoid unexpected failures not predicted by preclinical data, such as due to toxicities. Furthermore, the new candidate therapies can be ready for clinical trials quickly. The B-RAF^V600E^ inhibitor dabrafenib has already undergone clinical trials and is FDA-approved for the treatment of melanoma [25,26] and other human diseases [27,28,29]. Recently, accumulating evidence has suggested that multi-targeting kinase inhibitors, including dabrafenib and vemurafenib, used as anti-cancer drugs, also display anti-necroptotic effects [60]. Notably, the function of dabrafenib as a RIPK3 inhibitor was detected through the drug repositioning approach [30]. Previous studies have also confirmed that dabrafenib can selectively inhibit RIPK3 in various disease models in vitro and in vivo [30,31,32,33]. The findings of the current study have confirmed that the B-RAF^V600E^ inhibitor dabrafenib actually inhibits RIPK3-mediated necroptosis in the injured spinal cord and exerts a neuroprotective effect. Thus, the present study suggests that the administration of dabrafenib may be a promising candidate for the treatment of human SCI.

There are several limitations in the present study. First, the current study did not perform in vitro analyses to confirm the biological effect of dabrafenib on the spinal neurons or other neuronal cells. Several previous studies demonstrated that dabrafenib actually inhibits the RIPK3-mediated phosphorylation of MLKL in vitro using human melanoma cells, human dermal fibroblasts, human colon cancer cells, and leukemia cells [30,33,53]. However, the actual molecular mechanism of dabrafenib treatment in various neuronal cells obtained from the spinal cord remains to be fully elucidated. Second, the administration of dabrafenib might affect to other molecular pathways to regulate neural tissue damage and functional outcomes following SCI. Therefore, it is important to confirm that another SCI model lacking RIPK3 (e.g., RIPK3 knock-out) does weaken the case for RIPK3-mediated necroptosis attenuation by dabrafenib. In addition, recent studies have demonstrated that dabrafenib can attenuate the upregulation of inflammatory biomarkers, including TNF-α [61,62]. These studies imply that dabrafenib might inhibit the upregulation of TNF-α and consequently attenuate the RIPK3-MLKL signaling in the injured spinal cord.

## 5. Conclusions

We observed that dabrafenib administration in the acute phase significantly inhibited RIPK3-mediated necroptosis and reduced secondary neural tissue damage, such as demyelination, neuronal loss, and axonal damage, after SCI. In addition, we found that the neuroprotective effect of dabrafenib dramatically enhanced the recovery of the locomotor and sensory functions following SCI. Furthermore, the electrophysiological test objectively confirmed that the functional recovery was significantly enhanced by dabrafenib. These findings provide evidence suggesting the B-RAF^V600E^ inhibitor dabrafenib attenuates RIPK3-mediated necroptosis to provide a neuroprotective effect and promotes functional recovery after SCI. The administration of dabrafenib may be a novel therapeutic strategy for treating patients with SCI.
